# Facilitating Informal Support Among Older People Through Community-Based Initiatives: Identifying Underlying Mechanisms

**DOI:** 10.1093/geront/gnaf070

**Published:** 2025-02-15

**Authors:** Wenran Xia, Martina Buljac-Samardžić, Robbert Huijsman, Jan Smelik, Jeroen D H van Wijngaarden

**Affiliations:** Erasmus School of Health Policy and Management, Erasmus University Rotterdam, 3000 DR Rotterdam, The Netherlands; Erasmus School of Health Policy and Management, Erasmus University Rotterdam, 3000 DR Rotterdam, The Netherlands; Erasmus School of Health Policy and Management, Erasmus University Rotterdam, 3000 DR Rotterdam, The Netherlands; Nederland Zorgt Voor Elkaar, 3951 LC Maarn, The Netherlands; Erasmus School of Health Policy and Management, Erasmus University Rotterdam, 3000 DR Rotterdam, The Netherlands

**Keywords:** Active aging, Caregiving – Informal, Home and community-based care and services, Social support, Volunteering

## Abstract

**Background and Objectives:**

In the context of accelerated global aging and increasing insufficiencies in long-term care delivery, older people are encouraged to provide informal support to each other within their communities. However, the mechanisms facilitating such informal support among older people remain unclear. This study aims to address this gap by investigating the perspectives and experiences of various stakeholders involved in initiatives aimed at stimulating informal support in the community.

**Research Design and Methods:**

A qualitative multiple-case study was conducted in five Dutch initiatives stimulating informal support. Semistructured interviews were conducted with a total of 23 different stakeholders and relevant documents were analyzed. An abductive thematic analysis approach was used for data analysis.

**Results:**

Our analysis shows that community-based initiatives stimulate mutual support among older people by providing a coherent set of activities and facilities that indirectly, through community building, and directly influence individual behavior. On the community level, initiatives strengthen social cohesion, in terms of, for example, shared values and feelings of belonging. On the individual level, initiatives create opportunities to provide support, help individuals to recognize and strengthen their abilities to give support and stimulate individual motivation.

**Discussion and Implications:**

Our findings underscore the need for policies that support informal care through complementary processes, which work in tandem with formal care systems. Policies and practices taking the identified mechanisms into account are likely to stimulate older people to provide informal support to each other in the community, thus enhancing aging in place.

Accelerated global aging and cutbacks in healthcare budgets have increased pressure on healthcare delivery systems worldwide (Dieleman et al., 2016). Many countries advocate increasing reliance on informal support as a strategy to cope with insufficiencies in long-term services and support (Pani-Harreman et al., 2021). Informal support among older people is also seen as a way of stimulating active aging. Informal support refers to the assistance provided by those outside formal organizations and within one’s personal network and relationships (Lipman & Longino, 1982). It encompasses highly heterogeneous activities in the community setting from personal care to daily help such as transport (Rutherford & Bu, 2018; Van Den Berg et al., 2004). In this study, we employ a broad definition of informal support that covers all informal helping behaviors including informal care and volunteering (Siira et al., 2020, 2022), but we exclude informal help within one’s own family.

In recent decades, many European countries have advocated the perspective of active aging, which posits that community-dwelling older people should be viewed as valuable social capital and have the right to keep participating and contributing to the community (Foster & Walker, 2015). Facilitating and encouraging older people to support each other provides them with better opportunities to stay active and involved, live independently, and postpone institutionalized care (Rudnicka et al., 2020). Furthermore, active involvement of older people may also help to deal with shortcomings in long-term care services and support. Examples of initiatives and projects aimed at stimulating mutual support are the “time bank” initiatives in the UK (Simon & Boyle, 2008), the “Village” (Scharlach et al., 2012), and “NORC” (Naturally Occurring Retirement Community) programs in the United States (Greenfield, 2016). Although some programs were not developed specifically for older people, older people have become the vast majority of participants, which has led to interest from gerontology-related research (Greenfield, 2016). Despite the variations in type, size, and form, one of the common features among these initiatives is that they all encourage people to make use of their own resources and support each other while aiming to enable older people to stay in the community as long as possible in order to achieve better “aging in place.” In the Netherlands, the Social Support Act (WMO) was first introduced in 2007 and expanded in 2015 to distribute care and support responsibilities to municipalities and communities (Berkers et al., 2021). One of the core principles of the WMO is that residents need to increase their reliance on informal support from their personal social network. Within this context, a national platform called “Nederland Zorgt voor Elkaar (the Dutch care for each other)” was launched to unite 1,500 Dutch citizens’ initiatives focusing on the field of welfare, care, and living, aiming to stimulate mutual help among community-dwelling older people in the Netherlands.

The social-ecological model is one of the most widely used frameworks in exploring the role of the community context in informal support behavior among older people (Greenfield, 2012, 2016). According to the social-ecological theory, individuals are embedded in the context where multiple levels of environmental characteristics are nested. Therefore, individuals’ behavior is not only determined by their personal characteristics but also depends on the dynamic interplay between individual factors and environmental factors (Greenfield, 2012). The concept of “community gerontology” further develops this perspective by introducing the mesolevel, which is represented by the community/organization, as a critical bridge between macrolevel policies and microlevel individual factors in gerontology in the community (Greenfield et al., 2019). By focusing on the mesolevel, community gerontology provides a lens to examine how community organizations interact with individuals and the community to foster a supportive environment for aging in place.

Numerous studies have investigated the beneficial effect of providing informal support for older people, such as improving well-being and reducing depression (Murayama et al., 2021; Xia et al., 2024). Nevertheless, less is known about the factors at multiple levels that facilitate older people’s participation in informal support, especially regarding the underlying mechanisms that encourage older people to provide informal support to community members (Hou & Cao, 2021). On the individual level, studies have mainly focused on the motivation for informal support provision (Hansen & Slagsvold, 2020; Kramer et al., 2021; Same et al., 2020; Zarzycki & Morrison, 2021). These studies show that, on the one hand, older people are motivated intrinsically, where individuals are motivated by an inherent, internalized drive to provide support. On the other hand, people can be extrinsically motivated by external pressures, instrumental rewards, or social values (Zarzycki & Morrison, 2021). Few studies focus on individual factors other than motivation. Some studies have found that social demographic factors were relevant for older people’s willingness to participate in mutual informal support. For example, females are found to participate more in providing informal support due to different cultural gender expectations compared to males (Zygouri et al., 2021). Perceived support from one’s social relationships also matters (Inagaki & Orehek, 2017). Furthermore, community-level factors are important in explaining the mechanisms that facilitate older people’s provision of informal support to community members, especially support given outside the family. For example, Greenfield (2016) investigated a community-based program and found that neighborhood support was strengthened by the program. A strong social network seems to be relevant because individuals can be invited by network members to contribute (Lu et al., 2021). In addition, living in a community that is safe and resourceful, and has a strong sense of community among residents was associated with more informal support provision (Lu et al., 2021). Although studies have investigated the effects of individual and community factors on informal support among community-dwelling older people, the mechanisms through which higher-level factors influence individual-level factors, leading to informal support, remain underexplored.

## Current Study

The aim of this study is to explore how and why community-based initiatives affect mutual support behavior among older people. We take a comprehensive view of different levels of factors and the connections between them by exploring the perspectives of different stakeholders in the context of initiatives that aim to facilitate mutual support in the community. We specifically investigate five initiatives in the Netherlands, guided by the following research question:

What are the underlying mechanisms through which community-based initiatives stimulate mutual support among community-dwelling older people?

## Method

### Study Design

This study is a qualitative multiple-case study aiming to explore the mechanisms that facilitate older people in supporting each other in the community. A multiple-case study allows for in-depth, multifaceted explorations of the experiences and perceptions of participants and is appropriate for research aiming to understand a complex social phenomenon that is enacted in diverse contexts (Stake, 2013).

This study was approved by the Ethics Review Committee of the Erasmus University Rotterdam (application number ETH2223-0417). All participants provided oral or written informed consent.

### Selection of Cases and Participants

We identified relevant initiatives through a national platform in which 1,500 Dutch initiatives are united, called “Nederland Zorgt voor Elkaar (the Dutch care for each other).” We set inclusion criteria for initiatives that: (1) focus on stimulating informal support in the community and (2) involve older people aged 65 and older in providing informal support to each other. In order to include comprehensive characteristics of these initiatives, we contacted the general coordinator of the platform, who is familiar with all the initiatives, to assist us with the case selection. We identified two important factors for case selection, one being the location of the initiative, which could be rural or urban, and the other being whether the initiatives develop in households where residents live in the same building(s) or in neighborhoods. The general coordinator assisted in identifying cases by providing the researchers with a list of recommended initiatives that met the criteria (see Supplementary Materials). A total of five initiatives were purposefully selected. The overall characteristics of initiatives are illustrated in Table 1. It is worth noting that some initiatives could not be categorized under a single factor. For example, Austerlitz Zorgt organizes both neighborhood-based informal support and household-based programs in the village. The final selection was checked and discussed with the general coordinator.

**Table 1.** Overview of Initiatives

Initiative	Rural/urban	Type of organization	Coordinator	Initiative type
Zorgcoöperatie Hoogeloon	Rural	Care cooperative	Yes	Neighborhood-based and household-based
Vitality Cooperative America	Rural	Care cooperative	Yes	Neighborhood-based and household-based
Buurtcoöperatie Apeldoorn-South	Urban	Neighborhood cooperative	Yes	Neighborhood-based
Austerlitz Zorgt	Rural	Care cooperative	Yes	Neighborhood-based and household-based
Humanitas Deventer	Urban	Residential care center	Yes	Household-based

Different stakeholders, including both formal (e.g., initiative board members or coordinators) and informal (older residents) support providers, were interviewed to understand their experiences with informal support among older people in the initiative. The general coordinator of “Nederland Zorgt voor Elkaar” assisted us by contacting the board member of each initiative, who further helped us engage with interview participants. For each selected initiative, a snowball strategy was used for recruiting participants. A total of 23 individuals participated in the study, formally in 21 interviews. Given that most initiatives in “Nederland Zorgt voor Elkaar” are located in small cities or villages, participants in this study are primarily White and local Dutch residents. The background information of participants is presented in Table 2.

**Table 2.** Background Information of Participants

Respondent	Age	Gender	Role	Initiative
1	60–65	Male	Coordinator	A
2	70–75	Male	Resident	A
3	75–80	Male	Board member	A
4	80–85	Female	Resident	B
5	65–70	Female	Resident	B
6	70–75	Female	Board member	B
7	70–75	Female	Resident	B
8	60–65	Female	Coordinator	B
9	70–75	Male	Resident	B
10	65–70	Male	Board member	C
11	55–60	Female	Coordinator	C
12	70–75	Female	Resident	C
13	85–90	Female	Resident	C
14	50–55	Female	Coordinator	D
15	70–75	Male	Board member	D
16	70–75	Female	Resident	D
17	65–70	Female	Resident	D
18	65–70	Male	Board member	E
19	65–70	Male	Board member	E
20	50–55	Female	Coordinator	E
21	70–75	Female	Resident	E
22	70–75	Male	Resident	E
23	50–55	Female	Board member	E

### Data Collection

**Figure 1.**
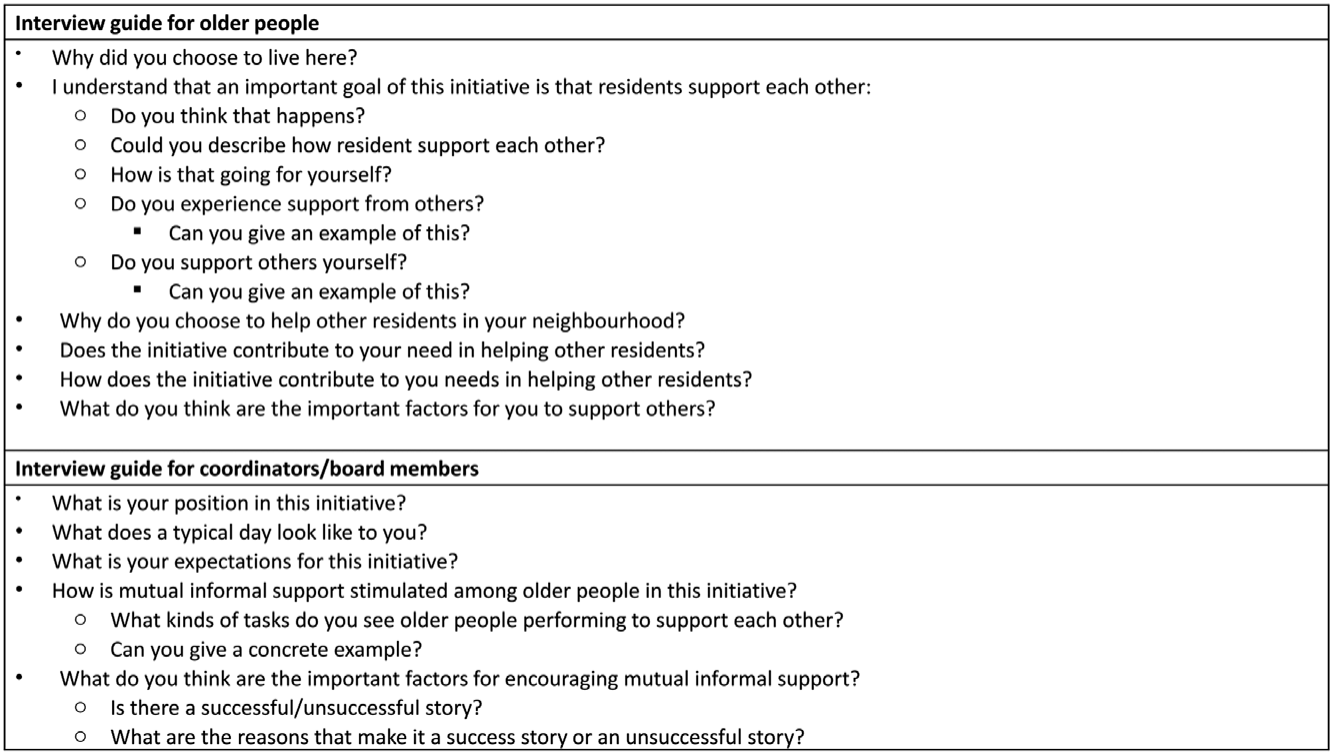
Abbreviated interview guide.

Semistructured interviews were conducted for this study. Interview data were collected by two Dutch-speaking researchers either in person or online via Teams between March and May 2023. For in-person interviews, participants were invited to share their views in a quiet and comfortable space they were familiar with. Questions were asked to older people, coordinators and board members, based on separate semistructured interview guides (Figure 1). Additional probing questions were asked based on the response of participants to elicit further details. Each interview took approximately an hour and was recorded digitally.

### Data Analysis

We employed an abductive thematic analysis approach to address the research questions, which is a hybrid process of inductive and deductive reasoning (Tavory & Timmermans, 2014; Thompson, 2022). This method allowed us to intertwine empirical data and theoretical frameworks from the literature; thus both parts amplify each other. Transcripts were first familiarized by reading them and identifying meanings and issues of potential interest in the data. Researchers then identified codes based on the data, through an open inductive process. This approach enabled an open analysis and avoided missing expected results. Next, overarching themes were identified by combining codes. We deductively used sensitizing concepts and theories derived from the literature, such as intrinsic and extrinsic motivations and social-ecological framework and intertwined these concepts with the empirical observations. Meanwhile, researchers inductively moved back and forth between data and literature to rethink existing theories and to unravel aspects not covered by literature, keeping our analysis open for surprising findings. Finally, themes were reviewed and refined by analyzing if the codes for each theme fit together and if they capture the entire data set. Themes and their names were then discussed and refined until consensus was reached. Development of themes and examples quotes are provided in Supplementary Table S2. Available materials from the initiatives such as their website and work documents provided by the initiatives were also used to help the researchers develop a better understanding of them. Nevertheless, the interview transcripts are the primarily data source, given their richness in capturing personal experiences and perspectives.

Interviews were audio-recorded and transcribed by Dutch interviewers for initial analysis. Transcripts were then translated into English for analysis. Data were imported into ATLAS.ti 23 software for initial coding and analysis (Hwang, 2008). The first author analyzed interviews and then collaboratively checked and completed the analysis with another author (J. D. H. van Wijngaarden) to enhance the trustworthiness of the study (Hennink et al., 2020). In addition, regular meetings within the research group were held to discuss codes and the delimitation and redefinition of themes as well as theoretical grounds. Furthermore, comparison of results based on Dutch and English transcripts showed good reliability of the results.

## Results

Among the five selected initiatives, three initiatives (Zorgcoöperatie Hoogeloon, America Left, and Austerlitz Zorgt) are located in rural areas, and two (Humanitas Deventer and Buurtcoöperatie Apeldoorn-South) are in urban cities. The initiative of Humanitas Deventer was developed in the household, while the rest were mainly developed in the neighborhood. All initiatives organize both informal and formal support for older residents in the community. Importantly, older people are deeply involved in volunteer work in all initiatives. Detailed information about the initiatives is shown in Supplementary Materials.

Our data analysis indicates that older people are facilitated to participate in informal support when they have the motivation and abilities, and when opportunities are provided. In addition, these individual-level mechanisms are strengthened by factors on the community level, and resources provided on the organizational level. It should be noted that there is much interaction between these levels, making it difficult to strictly discriminate between them.

### Individual-Level Mechanisms

#### Motivation

Many participants mentioned that they were primarily motivated to participate in informal support by the fulfillment they get from helping others (altruism), which means they decided to provide support based on empathy and concern for others:

I just do a lot of volunteer work here. I like it. I’ve always done it and I like it. And I see that people are happy about it. And then you will be happy yourself. That’s just very true. (Resp 21, resident of initiative E)

Another related motivation participants mentioned repeatedly was being useful and feeling meaningful to society, as they want their lives to have a purpose:

That makes me happier. Honestly. It really makes me happier. To also be able to mean something to someone else. (Resp 20, coordinator of initiative E)

For some participants, altruism alone may not be sufficient to take action. The combination of altruism and the enjoyment of doing the support task itself creates a win-win situation. One participant noted that her husband’s motivation for driving neighbors stemmed not only from his desire to help others but also from the personal enjoyment he gets from driving:

Well, then he says, first of all, I like driving. And driving someone from A to B doesn’t take me much time. It is scheduled…doing something good. But in a win-win. That he can do something he likes. Otherwise he won’t do it. (Resp 6, board member of initiative B)

In addition, reciprocity plays a role in motivating older people to participate. This reciprocity covers person-to-person relationships as well as the relationships between individuals and the environment in which they live. The motivation of reciprocity is represented as both delayed reciprocity and preparatory reciprocity. Delayed reciprocity refers to individuals attempting to return what they have received in the past. Some participants believe that they have received support in the past, and they therefore want to give back to others in return:

I was receiving benefits, and I couldn’t stand receiving benefits without doing anything in return. (Resp 13, resident of initiative C)

Some participants referred more to the concept of preparatory reciprocity, which is based on the belief that what they have provided may have a positive influence on satisfying their future needs:

What I just said, to still think of if we do it, maybe others will do it for us too…I don’t actually need it yet, but I think if necessary I can fall back on them. I do have that feeling. (Resp 4, resident of initiative B)

### Ability

To provide informal support not only requires motivation but also the perception of having relevant abilities to support others. Different respondents mention how they realized that their skills and abilities might be relevant (and fun) to support others:

I come from an education background and I’ve given and developed many courses during my work. And I simply enjoy it. So helping people, it’s in an area that I’m proficient in. (Resp 9, resident of initiative B)

It is worth noting that a skill does not only have to relate to a past profession. Daily skills such as cooking or reading newspapers are sufficient to help:

Well, I like reading the newspaper, because if I can read the newspaper to a lady who can no longer read, I can do that, so to speak. (Resp 10, board member of initiative C)

### Opportunity

Personal motivation and ability may not be sufficient on their own to initiate helping others. Actions can be greatly facilitated when people experience opportunities to engage in informal support, including having available time, social connections, and experiencing an open and inviting atmosphere to give support.

Most respondents mentioned that after retirement, they had time to participate in support activities:

Well, they are on the one hand, they are on average mostly elderly people, so also people who have that time, who also in part maybe make this kind of investment. (Resp 7, resident of initiative B)

Respondents also stated that they are more able to initiate support when they are socially connected. When they know others and meet others regularly it is easier to recognize the need for support, and also to offer it. In other words, being socially connected helps to align needs and support resources:

You’re more in touch with each other and then you also see more of what’s going on and then you also care sooner. Yes. If someone needs help you can also jump in together. (Resp 4, resident of initiative B)

However, respondents emphasized that these social connections and the provision of support should not be forced. People should feel invited and free to participate.

You can’t force people to make contacts with others, but there are plenty of opportunities, very accessible, for people to make contacts and access services and activities. (Resp 8, coordinator of initiative B)

### Social Cohesion in the Community

#### Trust relationships

Giving informal support is not only related to individual characteristics, our analysis shows, but also to characteristics of the community, specifically related to social cohesion. As a cognitive component of social capital, social cohesion is in the literature conceptualized as the collective community-level characteristic that puts an emphasis on norms, trust, and social bonds within the local social structure (Fone et al., 2007; Kawachi & Berkman, 2000). Participants mentioned several characteristics of the community related to social cohesion. First of all, they referred to the importance of trust relationships in the community. According to the residents, trust entails that neighbors are reliable and can be counted on when needed. By treating each other with kindness and respect, trust relationships can be enhanced among residents in the community, which facilitates the motivation to provide support:

I’ve needed little or no help so far. But I also know, if I needed help…The neighbor next door, he comes faithfully, if I tell him about that boiler, then a filter has to be replaced every now and then…Here my neighbors who are also very nice, have a key to my house. (Resp 16, resident of initiative D)

#### Feeling of belonging

Residents also mentioned the importance of feeling of belonging to the community. It refers to the feeling of being part of the larger group, which is the community. The feeling of being a member of the community enhances their motivation to contribute to the community:

And (older people help) precisely because you build a kind of community together, people regain their energy, or feel seen again, or feel useful again, or feel appreciated again. And you shouldn’t underestimate what that means, especially for older people, who were somewhat isolated. How people can flourish by indeed being part of a community again. (Resp 1, coordinator of initiative A)

#### Feeling of safety

Residents are also more willing to help when they feel safe in the community. Participants mentioned safety in terms of both physical environment and relationships with other residents in the community. On the one hand, a safe community enables residents to go out, creating opportunity to build more connection:

You are safe with each other. I feel that very strongly…I feel safe in community, don’t you? It is, people are nice to each other. I’ve never had anyone come across as unkind to me. Never. And then my sister says, what are you doing walking alone in the woods. But you can. Yes, it is possible here. (Resp 16, resident of initiative D)

On the other hand, the feeling of security provides opportunities to build tight relationships in the neighborhood:

I feel absolutely safe, because rather a good neighbor than a distant friend. Well, they had my key of course. (Resp 20, coordinator of initiative E)

#### Shared values

Last, it is important for residents to have a shared value of doing things together with others in the community, which means an awareness to come together as a group to bring positive change in the local community among residents is needed. It stimulates the obligation to contribute and motivates people to provide support to each other:

We enjoy getting things done for our village. Many people feel the same way. So, that makes the village strong. A powerful community. (Resp 12, resident of initiative C)

In general, a cohesive community helps to build an environment in which older people are encouraged to help each other. This cohesive atmosphere within the community makes individuals feel comfortable in the community, strengthens their motivation to help, and also creates opportunities.

### Facilitating Resources for Informal Support Provided by the Initiatives

All initiatives in our study provided facilitating resources that help to build a supportive environment to strengthen social cohesion in the community and stimulate motivation, abilities, and opportunities of older people to support each other. These include a central meeting place, coordinators who help to build support networks, a bottom-up governance structure, an information-sharing platforms, and both intra- and interorganizational collaborations.

#### A central meeting place

Participants from all initiatives emphasized the importance of a meeting place that provides physical spaces for community activities, which creates the opportunity for older people to get to know each other and build social connections:

If you zoom out for a moment, you can see a precautionary circle between people in the neighborhood, but you can also say, yes, but there must also be a central place that can be connected to. (Resp 23, board member of initiative E)

Importantly, the physical places help to build a group atmosphere among residents, enhancing a sense of community:

Here in (Initiative A) there are more of these kinds of what we call meeting places…And that also creates a piece of community, but we are a meeting place purely (for our initiative) “South comes together”. So there’s also a real “South comes Together” atmosphere. (Resp 1, coordinator of initiative A)

#### Coordinators

Participants from all initiatives emphasized the importance of a coordinator. Coordinators are sometimes called “village supporters” or “neighborhood assistants,” and work as the spiders in the community network. In most cases, coordinators are employees that receive an allowance from the municipality. However, they usually live in the same neighborhood and also work as volunteers in the communities. By getting in touch with residents and inviting them to the meeting place, coordinators help to build social connections among the residents, which facilitates the opportunities for mutual support among older people:

They (coordinators) keep an eye out for individuals who might fall through the cracks. For instance, there are quite a few elderly folks who withdraw and don’t open their doors anymore. The neighborhood assistant keeps an eye on that. Then, they’ll knock on the door, establish contact, and encourage them to come to the community center. And the most important thing is that they help re-establish relationships among the community. (Resp 3, board member of initiative A)

In addition, by visiting households and having daily talks with the residents, the coordinator recognizes residents’ available skills and talents as well as residents who are in need, and thus provides opportunities for matching support demand and supply:

The neighborhood assistants (coordinators) match people up…the neighborhood assistants say well, I know someone else and let’s go and have a coffee with them and get acquainted and if it clicks then I’ll let that go again…And then there really was that neighborhood assistant, who was indeed the intermediary and who could say, hey, supply and demand together, so to speak. That wouldn’t have happened if that neighborhood assistant hadn’t walked around there, because those people live close to each other, but they didn’t know anything about each other. (Resp 1, coordinator of initiative A)

A common and important quality of coordinators is that they are familiar with the community and work deeply embedded in the social network of the community. This quality enables them to get access to both the demand and supply side of support, and schedule support resources at the right place and the right time:

Well, the most important quality of a village supporter is that he knows the village like the back of his hand…if a lady is bored out of her mind that he has the opportunity to find someone else to become a walking buddy. And they have to be literally and figuratively embedded in the village. So he arranges bouquets of flowers and just goes to visit elderly people. (Resp 10, board member of initiative C)

#### Bottom-up governance structure

Besides places and coordinators, a bottom-up governance structure is also important. In each initiative, there is usually a board that takes charge of the management of suborganizations and day-to-day tasks. Board members in these initiatives are usually older residents who live in the community. Notably, most of them are also active informal support givers and contribute as volunteers. In most cases, such as Hoogeloon and Apeldoorn, coordinators are also board members. Older people who provide informal support are involved as active members of the suborganizations within the initiative such as transport group and walking buddy project. This bottom-up governance structure helps to establish trust relationship between residents and organization of initiatives, and helps to foster a greater sense of community.

You really have to come from the bottom up…So there really will have to come from the village, from below, from the demand, from the willingness of, it really will have to be picked up. (Resp 11, coordinator of initiative C)

#### Information-sharing platforms

Initiatives also provide platforms for information sharing in the community. In America, for example, there is an app called “Sido” on which residents can share information. Similarly, Hoogeloon has a newsletter as well as the village website, where the dynamics of the villages are very accessible for residents. These information-sharing platforms enable older people to have an insight into the ongoing affairs and dynamics in the community, which increases the opportunities for informal support directly. Furthermore, staying consistently informed of community affairs enhances the feeling as a group and belonging to the community. As one participant mentioned:

And of course we have the initiative app, everyone throughout the village exchanges things with each other. I have a cushion left, can someone use that, I have a pair of shoes in that size left…And then actually quite always a lot of people participate. (Resp 17, resident of initiative D)

#### Collaboration within and across organizations

Collaboration within and across organizations helps to provide a supportive environment for informal support. These collaborations provide opportunities for residents to learn skills and participate in the community based on their abilities by pooling resources such as training opportunities. Buurtcoöperatie Apeldoorn-South, for example, consists not only of a coordinator and meeting place but also an internship company and “neighborhood academy” that offers training for residents to learn skills (abilities) they can use to support others. Additionally, collaborations with formal care providers create a more integrated support systems, which could be perceived as community resources by residents, which helps to build a stronger sense of community. The America Left Cooperative, for example, runs in conjunction with the internal project “‘t Laefhoes” and has signed a contract with the external care organization called “Veil.” By doing so, informal support givers can collaborate with formal support professionals to provide support to those in need:

I’m a village team together with the district nurse, and if there is something wrong in the field of care, not just welfare, I ask her if she wants to take a look. That way I keep very short lines. She has her own care agency, home care, guidance, things like that. (Resp 14, coordinator of initiative D)

It is equally important to ensure clear responsibility boundaries during collaboration. Clear responsibility boundaries can prevent support givers from being overburdened due to providing support, and help preserve the motivation for sustainable participation:

And I’ve also noticed in all the volunteer work I’ve done, they always rely on people who are already doing volunteer work. And then you overburden them and they say stop. (Resp 6, board member of initiative B)

## Discussion

In this study we identified the underlying mechanisms through which community-based initiatives stimulate mutual support among community-dwelling older people, by abductively analyzing perceptions of different stakeholders in five Dutch citizens’ initiatives. Although numerous studies have explored the motivation and outcomes of providing informal support among older people and the role of the environment (Lu et al., 2021; Xia et al., 2024), fewer studies have focused on the mechanisms comprehensively. Our findings reveal that older people are facilitated to provide informal support to community members through a dynamic interplay of factors at the individual, community, and initiative level. At the individual level, older people are stimulated to provide informal support when they have and are aware of relevant abilities, are motivated, and experience the opportunity to participate in informal support. Social cohesion at the community level strengthens these individual factors, while community-based initiatives contribute by providing a bundle of activities and facilities that stimulate both the individual and the community level.

Consistent with other studies (Lu et al., 2021; Zarzycki & Morrison, 2021), we found that on the individual level older people in our study are driven to participate in informal support by the intrinsic motivation of the enjoyment of tasks and helping others (altruism) and by extrinsic motivation such as reciprocity and various forms of rewards. We also found that experienced ability and opportunity can be regarded as important factors at the individual level. Having relevant abilities and the awareness of being capable can benefit one’s self-efficacy, meaning a belief in one’s capacity to bring positive outcomes (Ryan & Deci, 2000). Having time and connections with others provides opportunities to give informal support. These findings mirror the so-called AMO-model, which is mostly used in HR-research to explain through what mechanisms HR-practices may improve performance (Marin-Garcia & Tomas, 2016). This model posits that individuals’ performance is determined by their ability, motivation and opportunity to perform. Performance only occurs when all three elements are present, the level of performance is determined by the level of each dimension (Marin-Garcia & Tomas, 2016). Therefore, HR-practices aim to influence all three, by creating bundles of practices that reinforce each other. This model also seems relevant to explain why people give informal support and to understand how informal support can be stimulated through such bundles of activities and facilities, such as community activities and an accessible community center.

We found that several community-level factors may facilitate informal support, including the feeling of belonging, trust, shared values, and safety, which are seen as important elements of neighborhood social cohesion (Chan et al., 2006; Kawachi & Berkman, 2000). These factors are in several ways connected with the individual-level factors we identified. A feeling of belonging provides emotional support for residents to stay in the community. Trust within the community and the feeling of safety encourage older people to share their support resources without fear of exploitation. Having shared values and common visions for the community also foster a collaborative spirit. These factors help to create opportunities for individuals to engage with the community they live in, and thus enhance their intrinsic motivation to participate in the community. Previous research revealed a reciprocal relationship between social cohesion and volunteering (Davies et al., 2024). Nevertheless, the impact of social cohesion on informal support provision seems to be stronger than the reverse (Horsham et al., 2024). Our findings emphasize the importance of investing in social cohesion to facilitate informal support for community-dwelling older people. However, given that our study was conducted in communities with primarily White residents, these findings may not be fully generalizable to more diverse settings or to communities with higher proportions of historically marginalized groups. Research from the United States suggests that support exchange among minoritized groups members may be also influenced by other factors such as resistance, survival, which may make them hesitate to participate in community initiatives outside their primary community of belonging (Reyes, 2023). These dynamics may influence the motivation for informal support provision, as well as the effectiveness of initiatives in fostering social cohesion.

**Figure 2.**
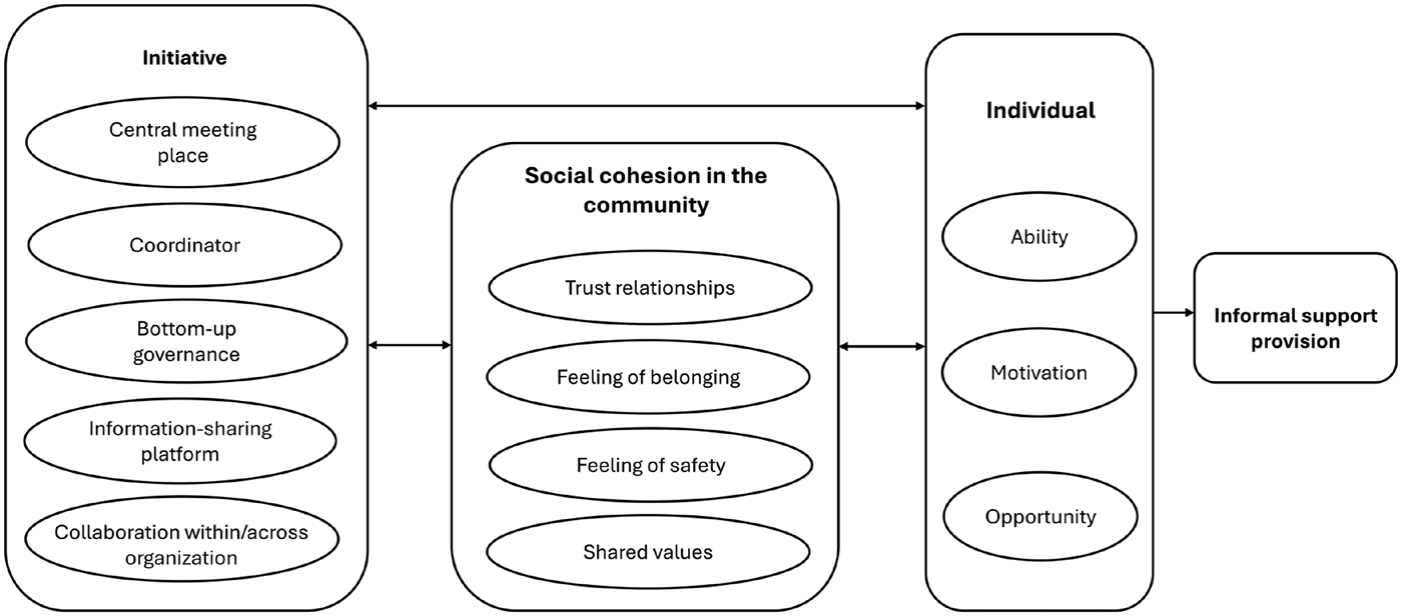
The visualized mechanisms facilitating older people to participate in informal support in the community.

We found that the initiatives in this study help to create a supportive environment for informal support by providing a bundle of activities and facilities that influence behavior through both direct and indirect mechanisms (as shown in Figure 2). Direct mechanisms include support arrangements organized by initiatives, such as coordinators who connect people and match informal support demands and supplies, providing opportunities for older people to support each other. Indirect mechanisms include promoting social cohesion within the neighborhood, thus facilitating larger interaction in the community. Both direct and indirect mechanisms involve the individual-level factors of motivation, ability, and opportunity to stimulate informal support. It should be noted that the relationships among factors at different levels can be bidirectional and nonlinear. Initiatives facilitate social cohesion in the community and individual factors, which in turn can promote further development of initiatives by enhancing community participation. These processes seem to reflect the social capital theory, which emphasizes the role of trust, network, and norm in the community (Kawachi & Berkman, 2000).

Initiatives not only help to create a supportive environment by encouraging more social interaction but also foster social capital in the community by enhancing trust relationships and feelings of belonging in the environment. Enhanced social cohesion increases the perception of being part of a larger group, and consequently stimulates individuals to participate in the collective by providing informal support to others. Results highlight the dual role of initiatives in stimulating informal support, both directly and indirectly through enhancement of social capital in the community.

Results of the current study reflect the social-ecological framework, which claims the role of factors on multiple levels, especially micro- and mesolevels in facilitating informal support and aging in place (Greenfield, 2012; Lu et al., 2021). Additionally, this study contributes particularly on two aspects. First, this study introduced a human resource management theory (the AMO-model), enriched with factors on individual level. Second, instead of listing factors on different levels separately as in most previous studies (Lu et al., 2021; Zarzycki et al., 2023), this study identified the underlying mechanisms that explain the relationships between different levels.

### Implications for Practice and Policy

Our findings may contribute to the design and implementation of policies related to aging and aging in place. Policymakers should be aware that bundles of practices are essential to facilitate informal support through factors on different levels. Improving older people’s abilities, enhancing their motivation, and providing opportunities for them to engage with the community should be considered simultaneously. In addition, interventions and community programs stimulating informal support are worth investing in, given they can help to build a cohesive environment and to enhance social connections, which can ultimately facilitate older people to take the action to participate in informal support.

It is worth noting that rather than advocating for complete substitution of public sector involvement, our findings underscore the need for policies that support informal care through complementary processes, which work in tandem with formal care systems. There is a concern that calls to promote informal support by older people themselves might shift the responsibility for care provision from the public sector to communities and individuals with insufficient resources, thereby perpetuating injustices (Martinson & Minkler, 2006). Informal support should not be seen as a substitute for formal care but rather as a way to enhance the overall support system. The initiatives we studied provided a supportive environment that complemented formal care services by building social cohesion and facilitating connections among older adults. This approach ensures that the burden of care does not fall solely on individuals or communities but is shared and supported through coordinated efforts. By investing in such initiatives and promoting policies that strengthen both formal and informal support structures, it is possible to avoid the potential pitfalls of devolution, thus creating a more equitable and effective care environment for older adults.

### Limitations

Several limitations need to be taken into account in this study. First, the older people acting as respondent were recruited from community organizations through initiative coordinators to maximize the feasibility. Older people who had less interaction with the community might therefore have been missed for the interviews. Second, whether the mechanisms apply to different contexts should be further investigated in future studies. We were not able to include participants with ethnic and migrant backgrounds, further studies could involve more ethnically diverse communities and participants. Despite these limitations, this study contributes to the topic of informal support in the community by deconstructing the mechanisms at and between the multiple levels in the context of community-level practice that enable older people to participate in informal support provision.

## Conclusions

Given the rapid shift of global demographics, the increasing pressure on the healthcare budget, and the sustained interest in age-friendly interventions in the community, clarifying the mechanisms of community-based informal support on both individual and community level is important. This study reinforces the importance of stimulating informal support by focusing on multilevel factors, including the combination of ability, motivation, and opportunity on the individual level, social cohesion on the community level, and facility support provided by initiatives on the organizational level. These findings are essential for further development of practical efforts to promote neighborhood informal support in the community, thus fostering aging in place. Empirical findings of this study support the idea of environmental gerontology and emphasize the beneficial role of community organizations in facilitating individual support behavior, which can be referred to by policymakers and practitioners.

## Supplementary Material

gnaf070_suppl_Supplementary_Material

## Data Availability

Data are unavailable due to ethical issues associated with the qualitative research design. This study was not preregistered.
