# Supplementary material for: Facilitating Informal Support Among Older People Through Community-Based Initiatives: Identifying Underlying Mechanisms
Source: Gerontologist. 2025 Feb 15;65(6):gnaf070. doi: 10.1093/geront/gnaf070 (PMC12086058; doi:10.1093/geront/gnaf070)
Supplement: gnaf070_suppl_Supplementary_Material [file gnaf070_suppl_supplementary_material.docx]

**Online Supplementary Material**

**Supplementary Table 1**. Characteristic dimension for initiative selection.

|  | **Rural** | **Urban** |
| --- | --- | --- |
| **Neighborhood-based** | - [Zorgcoöperatie Hoogeloon](https://zorgcooperatie.nl/) - Vitality Cooperative America Left | - Buurtcoöperatie Apeldoorn-South |
| **Household-based** | - Austerlitz Zorgt | - Humanitas Deventer |

**Supplementary Table 2.** Themes and Example Quotes from the Interviews.

| **Levels** | **Secondary themes** | **Primary themes** | **Initial codes** | **Sample Quotes** |
| --- | --- | --- | --- | --- |
| Individual | Motivation | Altruism | Help for make others benefited | *“To help and support others. And by helping others, you see that someone else's situation improves…So, it's an inner drive to do something for others, to help others. That's where the motivations lie.” (Resp 12, resident of initiative C)* |
|  |  |  | Like seeing others happy | *“I just do a lot of volunteer work here. I like it. I've always done it and I like it. And I see that people are happy about it. And then you will be happy yourself. That's just very true.” (Resp 21, resident of initiative E)* |
|  |  | Being useful and feeling meaningful | Happy because help means something for others. | *“That makes me happier. Honestly. It really makes me happier. To also be able to mean something to someone else.” (Resp 19, board member of initiative D)* |
|  |  |  | Feels meaningful despite of disability | *"At the moment I'm disabled, I don't have any voluntary work so I notice that I'm disappointed that I can do less for society. So, with little things like this I kind of feel like I do something and mean something. And uhm.. that I'm not just surviving so to speak" (Resp 16, resident of Initiative D)* |
|  |  | Enjoyment of doing the support task itself | Help driving people because of the enjoyment of driving | *“I think that's more the intrinsic motivation than the enjoyment of driving itself…I simply enjoy driving. No doubt about it.” (Resp 9, resident of initiative B)* |
|  |  |  | Feel happy and active for performing the best | *“That's where I'm at my best I think. Yes, I enjoy it, it makes me happy, it makes me active.” (Resp 20, coordinator of initiative E)* |
|  |  | Reciprocity | It is important to have reciprocal relationships | *“I think it's just something... When you give something to someone, you also get something back. Yes. And if you only take, then you get.... Then you can't give anything back either. No. I just think it's important that there is a reciprocal relationship.” (Resp 11, coordinator of initiative C)* |
|  |  |  | preparatory reciprocity | *“What I just said, to still think of if we do it, maybe others will do it for us too… I don't actually need it yet, but I think if necessary, I can fall back on them. I do have that feeling.” (Resp 4, resident of initiative B)* |
|  | Ability | Realizing skills and abilities being relevant | Professional training facilitates to do things | *“I come from an education background and I've given and developed many courses during my work. And I simply enjoy it. So helping people, it's in an area that I'm proficient in.” (Resp 9, resident of initiative B)* |
|  |  |  | Help reading papers because being able to | *“Well, I like reading the newspaper, because if I can read the newspaper to a lady who can no longer read, I can do that, so to speak.” (Resp 10, board member of initiative C)* |
|  | Opportunity | Time | Having time after retirement | *“Most of the volunteers I have are of course the elderly. These are retired people who suddenly have a lot of time and they really enjoy doing volunteer work.” (Resp 14, coordinator of initiative D)* |
|  |  |  | Older people have time to make investment | *“Well, they are on the one hand, they are on average mostly elderly people, so also people who have that time, who also in part maybe make this kind of investment.” (Resp 10, board member of initiative C)* |
|  |  | Socially connected | Recognizing each other’s ability | *“People discover from each other that you have certain skills and dexterity in something and that people then say, well then, if you have a job to do I can help you. And indeed, the idea of people going shopping together. Yes. And so people who, just little networks of people who wouldn't have met otherwise are created. And who now get together and support each other.” (Resp 1, coordinator of initiative A)* |
|  |  |  | Connection facilitates awareness of needs | *“You're more in touch with each other and then you also see more of what's going on and then you also care sooner. Yes. If someone needs help you can also jump in together.” (Resp 4, resident of initiative B)* |
|  |  | Being invited and free to participate | Do because wants to do by oneself | *“Whether you want it or not, it won't work and it won't succeed. So that's easy. But if there are people who want to do it, then it will work. The most important thing is that there are people who do it because they want to.” (Resp 3, board member of initiative A)* |
|  |  |  | Voluntary basis facilitates participation | *"I think that's also because everything is on a voluntary basis, no one is forced to do anything.” (Resp 14, coordinator of initiative D)* |
| Community | Social cohesion in the community | Trust relationships | Respect builds trust relationship | *“By treating others with respect, you also build a form of trust in each other. And I think that's the basis of getting along well with each other, that people trust you.” (Resp 12, resident of initiative C)* |
|  |  |  | Mutual trust between residents | *“But trust is the most important thing. Look, they need to trust us that we won't do anything bad to them. And we need to trust them that they find it enjoyable and pleasant that we're doing it.” (Resp 5, resident of initiative B)* |
|  |  | Feeling of belonging | Feeling happy for being part of the group | *“They took me in and I'm happy to be part of it. That's how I feel.” (Resp 17, resident of initiative D)* |
|  |  |  | Belong through help | *"I'm glad that I've been doing this (helping) because, despite my visual impairment, I still feel like I belong." (Resp 13, resident of initiative C)* |
|  |  | Feeling of safety | Feeling safe living in the community | *"I say, I feel safe in Austerlitz, don't you? It is, people are nice to each other. I've never had anyone come across as unkind to me. Never. And then my sister says, what are you doing walking alone in the woods. But you can. Yes, it is possible here." (Resp 16, resident of initiative D)* |
|  |  |  | Feeling safe living with neighbors | *“Absolutely (feel safe), because rather a good neighbor than a distant friend. Well, they had my key of course. Another family, by the way. And when things weren't going well, I was allowed to call at night.” (Resp 20, coordinator of initiative E)* |
|  |  | Shared value of doing things together | Collective identity of togetherness | *“So there's also a real South does Together atmosphere… I think that is, certainly for a large group of people, one of the reasons they come to us and not somewhere else. Because they're just really part of that community.” (Resp 1, board member of initiative A)* |
|  |  |  | Achieve more by doing things together | *"Because often, you have people sitting in different places, doing different things. And when you work together, you can achieve more and spend less time setting everything up"* [*(Resp 6, board member of initiative B)*](https://go.atlasti.com/3dfa15a6-cc30-4c2e-a337-7c4d2c8fb2db/documents/13aeb7f9-d64b-46bd-b623-11ff4ffcb5f8/quotations/2ed515b6-1169-46f9-9ba4-4160fbdb7bfe) |
| Initiative | A central meeting place | Meeting place | Importance of the infrastructure to meet each other | *“You really need a place where you can meet. A village hall, a sports canteen for all I care. You must have that. That is an infra-structural condition. If you have such a place, yes then you have to organize activities. And that happens automatically because if you have a building, you don't want to leave it empty.” (Resp 15, board member of initiative D)* |
|  |  |  | Meeting places enhance the sense of community | *“Here in (the neighborhood) there are more of these kinds of what we call meeting places…And that also creates a piece of community, but we are a meeting place purely from Zuid doet Samen. So there's also a real South does Together atmosphere and it's a South does Together atmosphere.” (Resp 1, coordinator of initiative A)* |
|  |  | Central location in the community | Central location to aware support needs | *"And because we are all in the middle of the village, you know what is happening in the village and whether anything is needed.” (Resp 14, coordinator of initiative D)* |
|  |  |  | Central hub for community life | *“Everything happens from here, so it really is the central point. The gym, the school! The children also come here and parents also meet again in the schoolyard. This is really the central point." (Resp 14, coordinator of initiative D)* |
|  | Coordinators | Help building social connections | Help to build contact among residents | *“She (coordinator) says, can you do this or that. And that's how you get some contacts. I'm not the type to easily go anywhere...” (Resp 17, resident of initiative D)* |
|  |  |  | Building connections among resdients | *“The neighborhood assistants (coordinators) match people up. That wouldn't have happened if that neighborhood assistant (coordinator) hadn't walked around there, because those people live close to each other, but they didn't know anything about each other.” (Resp 1, coordinator of initiative A)* |
|  |  | Matching support demand and supply | Monitoring needs among residents | *“They keep an eye out for individuals who might fall through the cracks. For instance, there are quite a few elderly folks who withdraw and don't open their doors anymore. The coordinator keeps an eye on that. Then, they'll knock on the door, establish contact, and encourage them to come to the community center.” (Resp 3, board member of initiative A)* |
|  |  |  | Personalized matching demand and supply | *“So the one who hear the stories on the street and they actually have there and that is stored and it could be that someone else streets away who is having the same issue but on the demand side. And then the neighborhood assistants say well, I know someone else and let's go and have a coffee with them and get acquainted and if it clicks then I'll let that go again. …And then there really was that neighborhood assistant, who was indeed the intermediary and who could say, hey, supply and demand together, so to speak.” (Resp 1, coordinator of initiative A)* |
|  | Bottom-up Governance structure | Bottom-up Governance structure | Importance of bottom-up approach | *“You really have to come from the bottom up. You can't pick up something and copy it in another place and say, so this is it and start it. So there really will have to come from the village, from below, from the demand, from the willingness of, it really will have to be picked up.” (Resp 11, coordinator of initiative C)* |
|  |  |  | Bottom-up organization enables empowerment through ownership | *So really organised close to the people and from the bottom up. So that you really say, yes, residents regain some kind of ownership of their own environment.” (Resp 1, coordinator of initiative A)* |
|  | Information sharing platforms | Information sharing platforms | Informing the community through the newsletter | *“And one of the activities I'm also involved in is a village newsletter…You can put a lot of things in there. I also include information about dementia and all sorts of things. But by doing that, you inform people well, and it's really well read by the elderly.” (Resp 12, resident of initiative C)* |
|  |  |  | Use of APP promotes community exchange | *“And of course we have the Austerlitz app, everyone throughout Austerlitz exchanges things with each other. I have a cushion left, can someone use that, I have a pair of shoes in that size left...” (Resp 17, resident of initiative D)* |
|  | Collaboration within and across organizations | Internal collaborations | Collaboration within organization makes work effective | *“I focus on the bigger picture, setting the vision and so on. I also work closely with Sjan, and it works very well. We're quite different, but together, it's very effective.” (Resp 6, board member of initiative B)* |
|  |  | Cross-sectoral collaborations | Collaboration with the district nurse make efficient response to needs | *"I'm a village team together with the district nurse, and if there is something wrong in the field of care, not just welfare, I ask her if she wants to take a look. That way I keep very short lines. She has her own care agency, home care, guidance, things like that." (Resp 14, coordinator of initiative D)* |

**Overview of cases**

[**Zorgcoöperatie Hoogeloon**](https://zorgcooperatie.nl/)

The [Zorgcoöperatie Hoogeloon](https://zorgcooperatie.nl/) is a healthcare cooperative in the municipality of Hoogeloon in the province of North Brabant. The initiative was built since 2005 and was the pioneering care cooperative in the Netherlands. The cooperative has been providing various service for residents with various conditions from mild to severe within the village, including care, meals, accommodation and so on, based on voluntary work. In addition, there are two village supporters (coordinator) who works to help residents coping with various issues related to the community. There is an activity center in Hoogeloon, where daytime activities are organized by volunteers from the care cooperative and two professionals from the outside care organization. For meals, a cooking group made of volunteers cook meals for the older people who live in Hoogeloon every Tuesday. Except organizing social support, the cooperative collaborates with Joris Zorg, which is the professional care organization, with a professional healthcare team to provide home care for residents. In addition, there are two care villas in the village to care for older people with a diagnosis of dementia and an appropriate indication. By participating in the Joris Zorg client council, residents are able to exchange information with a client council representation with healthcare professionals are involved.

**Vitality Cooperative America Left**

America is a village located in the municipality of Horst aan de Maas in the province of Limburg, with the population of around 2,300 people. The America Left in America is a care cooperative aiming to promote a vital community and prevent professional care as much as possible. The America Left provide a platform to support America’s residents helping each other voluntarily. The cooperative organizes various activities include helping with tasks in and around the house, finding a buddy for daily activities such as walking or cycling, volunteer work, and consultancy of care-related questions. Activities are arranged with the coordination of two village supporters (coordinators). All board members of the cooperative are volunteers. Working together with professionals, volunteers in America Left provide care and welfare services as integrated as possible. Additionally, the 't Laefhoes in America is a meeting place for professional healthcare with various participants. In the building, there is a public living room, a kitchen and a garden for all residents of America and surrounding areas.

**Buurtcoöperatie Apeldoorn-South**

Buurtcoöperatie Apeldoorn-South is a neighborhood cooperative located in Apeldoorn, a city located in the province of Gelderland with population of approximately 30,000 residents. Based on the background of the Social Support Act (WMO) of the Netherlands, where citizens and their personal network need to hold responsibilities for care, the cooperative has been established in 2013 to support mutual support for residents. It is an umbrella organization that consists of four distinct functional branches. The first branch is the neighborhood assistants (coordinators) who work in each neighborhood in Apeldoorn. The second branch is a meeting place called “Ons Honk”, where residents come to socialize and where day care services are provided. Besides, an internship company is running in the cooperative where people from ROCs (Regional Training Center) or students from social studies come for training. Last, there is a branch called “neighborhood academy” that offers courses or lessons to invite residents to participate. Additionally, there is a new branch has been built called “locomotive”, aiming to offer guidance to people who are re-entering the labour market.

Apeldoorn South was separated into several smaller neighborhoods. The neighborhood assistant (coordinator) was employed by the cooperative and works for each neighborhood.

**Austerlitz Zorgt**

Austerlitz is a village located in the province of Utrecht with about 1,800 residents. The Austerlitz Zorgt is a care cooperative in the village, where mutual informal support is village-wide implemented. The Austerlitz Zorgt was established in 2012, aiming to improve the quality of life and support independent living among residents. A village supporter (coordinator) to whom everyone can ask questions and support from neighbors in handling small tasks helped on the volunteering arrangements. With the coordination of the coordinator, various services for residents including shopping, transport, meal provision, garden maintenance, domestic help and so on, are organized. Almost all these services are provided by volunteers who are mainly older residents of Austerlitz. The organization also collaborates with professionals such as district nurse to provide healthcare services. In addition, there is a community center in the center of the village, where many groups and activities in which residents can participate are organized. One must pay dues and be a member of the Austerlitz Zorgt to participate. Notably, nearly all residents aged 18 and above are members.

Therefore, by listening to the needs of residents, more and more clubs are formed and their needs are met. Furthermore, initiatives such as the Austerlitz Rijdt (where a person drives someone somewhere) are based on voluntary work. More than 100 volunteers help with small tasks and support fellow Austerlitz residents through voluntary work.

**Humanitas Deventer**

Humanitas Deventer is a residential care center for older people with needs of support, located in Deventer, which is a city in the province of Overijssel. It is a household-based initiative consisting of three buildings. One is called Ludgerus which consists of 300 apartments and provides basic care. Only people who meet the profile can get such an apartment. Older people will move to a nursing home if they need day-to-day care, so that they can live independently. The other is an 11-storey unit, which has become a 55-storey or above unit since November of 2022. Here the Voorzorgcirkels initiative is in full swing to increase and stimulate mutual support. Finally, the main building of Humanitas Deventer organizes activities where residents can meet each other and build connections. Although there are also coordinators in the Humanitas Deventer, they are also the residents and do coordinating tasks voluntarily. A unique characteristic of Humanitas Deventer is that students can rent several apartments free of charge on the condition that they do their best to help the older people. By doing this, Humanitas Deventer integrates young people into their main building and creates a cross-generational building.
